# Exploring Neuropsychiatry: Contemporary Challenges, Breakthroughs, and Philosophical Perspectives

**DOI:** 10.7759/cureus.49404

**Published:** 2023-11-25

**Authors:** José Carlos Medina-Rodríguez

**Affiliations:** 1 Department of Neurology & Neuropsychiatry, National Institute of Psychiatry, Mexico City, MEX; 2 Postgraduate Studies Division, National Autonomous University of Mexico, Mexico City, MEX

**Keywords:** neuropsychiatry, neuroscience, psychiatry, neurology, evolutionary biology

## Abstract

This editorial provides a concise and updated overview of neuropsychiatry, emphasizing its definitional challenges and profound implications for education, training, research, and the integration of phenomenology and philosophy of mind.

Neuropsychiatry, situated at the crossroads of neurology and psychiatry, grapples with complex definitional issues that impede research progress. Establishing a unified conceptual framework is essential for focused research and delving into fundamental questions regarding topics like "consciousness."

Nevertheless, the integration of philosophical perspectives into neuropsychiatry, while valuable, faces hurdles due to conceptual ambiguities and the fluid boundaries of the field. These obstacles disrupt research and hinder progress in effectively addressing neuropsychiatric conditions.

This editorial advocates for a systematic approach to defining neuropsychiatry to mitigate these concerns. Additionally, as neuropsychiatry evolves, it necessitates an integrative approach. Recent advancements in neuroscience, propelled by technologies like artificial intelligence and advanced neuroimaging, reshape our comprehension of brain-behavior interactions, offering potential biomarkers and comprehensive treatment approaches.

## Editorial

Current state of neuropsychiatry

This editorial provides an insightful and up-to-date overview of neuropsychiatry, highlighting the field's dynamic nature and its significant impact on education, healthcare professional training, research, and the integration of philosophy.

In the quest to define neuropsychiatry, a field enriched by its integration of neurology and psychiatry, there lie exciting challenges and opportunities. This article explores how a clearer definition can propel research within the field forward. A unified conceptual framework for neuropsychiatry is not only beneficial but also necessary to direct focused research, paving the way for groundbreaking discoveries like biomarkers and a deeper understanding of complex phenomena such as "consciousness." Addressing current conceptual gaps promises to enhance training, equipping practitioners with the tools they need to navigate the interplay between neurological and psychiatric conditions effectively.

Furthermore, the incorporation of phenomenology and the philosophy of mind into neuropsychiatry presents a fertile ground for innovation. While there are hurdles due to the fluid nature of the field's boundaries, overcoming these challenges can significantly enrich our understanding of patients' subjective experiences and the nuances of mental disorders.

In research, establishing a consistent conceptual framework is a promising step towards unifying research efforts. Such a unified approach can facilitate the development of comprehensive theories and treatment methods, accelerating progress in comprehending and treating neuropsychiatric conditions effectively.

Thus, this editorial advocates for a structured and optimistic approach to defining neuropsychiatry. Such an initiative is expected to not only refine educational and training programs but also to invigorate research activities and enable a more holistic integration of philosophical viewpoints. These advancements are crucial for advancing patient care and deepening our understanding of the multifaceted nature of neuropsychiatric disorders.

Contemporary challenges

Foremost, developing a clear and accessible conceptual framework for neuropsychiatry remains a paramount goal. This interdisciplinary field, skillfully blending elements of neurology and psychiatry, has evolved significantly over the past century. It integrates insights and methodologies from diverse areas, such as behavioral neurology and biological psychiatry, evolving through rigorous debate and gradual refinement within the medical and scientific communities [1].

Further, recognizing the distinct nature of neuropsychiatry is crucial. This discipline requires a unique amalgamation of skills to adeptly navigate the complex interplay between neurological and psychiatric disorders. For medical professionals, particularly those in training, a thorough understanding of neuroanatomy and neurophysiology is vital [2]. This foundational knowledge is essential for the proficient use of advanced diagnostic tools in neuropsychiatric cases, which often demand a comprehensive understanding of the nervous system's anatomy, physiology, and circuitry to interpret complex psychiatric symptoms without resorting to reductionist approaches.

Moreover, integrating psychosocial sciences into neuropsychiatry is not just beneficial but essential. The role of cultural factors in neuropsychiatric conditions, often overlooked, deserves more attention, especially given the limited focus on these aspects in current research. Addressing this gap is crucial for a more holistic understanding of neuropsychiatric phenomena.

Therefore, the ongoing refinement and effective dissemination of neuropsychiatry’s scope are of utmost importance. Endorsing global efforts to educate professionals and stimulate research curiosity in these areas is essential. Such initiatives are key to unveiling the deeper aspects of the human experience. These efforts not only advance the field but also increase its visibility and understanding among healthcare professionals and the broader public. Well-defined neuropsychiatry is instrumental in highlighting its role in advancing medical science and improving patient care.

Topics of interest and modern advances

The extraordinary significance of recent advancements in neuroscience is undeniable, particularly with the integration of cutting-edge technologies such as artificial intelligence [3], advanced neuroimaging techniques, and brain stimulation therapies. These innovative tools, in synergy with advances in our understanding of neuroanatomy and neurophysiology, are reshaping our perception of the brain-behavior relationship. This transformative evolution offers the potential to generate robust data, which could be recognized as critical biomarkers in mental health, thereby marking a pivotal milestone in both neurology and psychiatry [4].

Similarly, the impactful advancements in psychoneuroimmunoendocrinology warrant close attention. This burgeoning field, focusing on the immune system's influence on psychiatric diseases, underscores the significance of the brain's active, multicellular immune system. The interplay between this system and peripheral immunity is instrumental in shaping behavior, thereby establishing critical areas of study within neuropsychiatry [5].

Delving deeper, there are numerous concepts suggesting a connection between immune dysfunction and neuropsychiatric diseases. For instance, the concept of allostatic load, which represents the cumulative 'wear and tear' on an organism from repeated adaptive responses to stress, has garnered renewed interest. Additionally, the impact of nutrition, especially personalized diets, on neuroendocrine modification is gaining traction. These dietary interventions could mitigate dysregulation in biological systems, often associated with aging, through potential anti-inflammatory or antioxidant mechanisms, thus influencing both brain and behavior [6].

However, consistent with the complexities of neuroscience, the exact molecular pathways through which immune cells and cytokines influence neuropsychiatric diagnoses are still elusive. Persistent questions regarding which brain regions are implicated, the nature of brain-immune interactions, and whether these predominantly occur within the brain or the periphery, remain unanswered [5]. Therefore, the exploration of these pathways is not just of significant interest but also essential for those striving to decipher this facet of neuroscience.

It is imperative to recognize that the pursuit of these goals requires a deep-seated curiosity for the unknown, a curiosity that fuels transformative advancements in the field. This transformation extends beyond the neurological or psychiatric aspects of complex cases, advocating for a holistic approach. Such an approach promises more precise and individualized treatment strategies.

Furthermore, the resolution of these complex issues calls for a substantial and indispensable philosophical contribution. Philosophical perspectives offer invaluable insights, particularly when grappling with the nuanced questions central to psychiatry and neuroscience. The role of philosophy is crucial in interpreting the implications of these potential biomarkers and in understanding the broader existential and ethical dimensions of mental health and human behavior. The integration of these philosophical viewpoints with scientific advancements is a crucial and promising step towards addressing some of the most challenging inquiries in neuropsychiatry.

Philosophical foundations of neuropsychiatry

This exploration, consciously avoiding an exhaustive debate and eschewing the reduction of the mind to a mere byproduct of nervous system structures, aims to underscore the vital contributions of philosophy to neuropsychiatry. These disciplines reveal valuable, yet frequently underappreciated, insights. Neuropsychiatry, rooted in the meticulous analysis of complex cases and their phenomenology, sheds light on the intricate interplay between neurology and psychiatry [1]. The goal here is to articulate clear connections between philosophical concepts and neuropsychiatric phenomena, utilizing illustrative examples for better understanding.

Take, for example, Daniel Dennett's insights into consciousness, which offer enriched comprehension of conditions such as non-epileptic seizures. Dennett's "multiple drafts" model, suggesting varied levels of awareness and processing in the brain, mirrors the diverse manifestations of consciousness seen in these conditions [7]. Philosophical perspectives on the physical basis of consciousness enhance our grasp of how specific brain regions, like the temporoparietal junction - pivotal for multisensory integration, self-awareness, and body image - relate to these conditions and contribute to the broader debate about the nature of "consciousness."

Extending beyond Dennett's theories, a range of philosophical schools could provide insightful perspectives on the foundational philosophy of neuropsychiatric disorders. However, delving deeply into these viewpoints is beyond the present discussion’s ambit. Moreover, this discourse encompasses the application of these philosophical insights to phenomena such as neglect syndromes and their links to fascinating neuropsychiatric conditions, including alien hand syndrome, delusions of denial, and autoscopic/heautoscopic experiences. While other significant examples are worthy of exploration, these cases are sufficient to illustrate how blending these disciplines can broaden neuropsychiatry's scope. A detailed examination of these issues may catalyze further research and breakthroughs across neuropsychiatry, mental health sciences, and psychosocial sciences.

Thus, it becomes crucial to recognize that these philosophical perspectives offer a distinctive framework for exploring and potentially reinterpreting neuropsychiatric phenomena. They encourage a more encompassing approach, one that integrates both the neurobiological and experiential dimensions of complex conditions.

Final remarks

Neuropsychiatry, positioned at the cutting edge of a dynamic and ever-progressing field, has witnessed significant strides thanks to recent advancements in neuroscience. These developments have profoundly enriched our understanding of the intricate interaction between the brain and behavior, shedding light on the neuroanatomical and physiological underpinnings of neuropsychiatric disorders. Yet, it is crucial to understand that these insights are not standalone revelations but part of a larger, interconnected picture.

An integrative approach, therefore, becomes indispensable. It's about weaving insights from psychosocial sciences and philosophy into the fabric of neuropsychiatry, creating a tapestry of comprehensive understanding. This holistic perspective is not merely advantageous; it is imperative. It serves as a lighthouse, guiding mental health research towards breakthroughs and kindling the curiosity essential for delving into the complexities of the human psyche.

By adopting this integrative approach, the field can move beyond a basic understanding of neuropsychiatric conditions. The objective is to refine diagnostic tools and tailor treatment plans that truly resonate with the unique experiences and needs of those grappling with these disorders.

In essence, the future trajectory of neuropsychiatry relies on its capacity to fuse the insights of neuroscience seamlessly with the wisdom of psychosocial sciences and philosophy. Such a unification promises to transform our approach to mental health care, potentially elevating the quality of life for those living with neuropsychiatric disorders significantly.
